# Association Between Zolpidem Use and Glaucoma Risk: A Taiwanese Population-Based Case-Control Study

**DOI:** 10.2188/jea.JE20140051

**Published:** 2015-01-05

**Authors:** Yi-Hao Ho, Yue-Cune Chang, Wei-Cheng Huang, Hsin-Yi Chen, Che-Chen Lin, Fung-Chang Sung

**Affiliations:** 1 Department of Ophthalmology, China Medical University Hospital, Taichung, Taiwan; 2 Department of Mathematics, Tamkang University, Taipei, Taiwan; 3 School of Medicine, Medical College, China Medical University, Taichung, Taiwan; 4 Management Office for Health Data, China Medical University Hospital, Taichung, Taiwan; 5 Department of Public Health, China Medical University, Taichung, Taiwan

**Keywords:** zolpidem, glaucoma, Taiwanese

## Abstract

**Background:**

To date, the relationship between zolpidem use and subsequent risk of glaucoma in a Taiwanese population has not been assessed.

**Methods:**

We used data from the National Health Insurance system to investigate whether zolpidem use was related to glaucoma risk. A 1:4 matched case-control study was conducted. The cases were patients newly diagnosed with glaucoma from 2001 to 2010. The controls were randomly selected non-glaucoma subjects matched by sex and age (±5 years). Zolpidem exposure and/or the average dosage of zolpidem used (mg/year) were evaluated. Medical comorbidities were considered as confounding factors. Multiple logistic regression models were used to evaluate the potential risk of zolpidem exposure on glaucoma with/without adjustment for the effects of confounding variables.

**Results:**

The exposure rate of zolpidem use in the glaucoma group was significantly higher than that of the control group (2.8% vs. 2.0%, *P* < 0.0001). The adjusted odds ratio (OR) of the risk of glaucoma for those with zolpidem use vs. those without was 1.19 (95% confidence interval [CI], 1.02–1.38). Compared to non-zolpidem users, zolpidem users with an average dose of more than 200 mg/year had significantly increased risk of glaucoma (OR 1.31, 95% CI 1.03–1.68).

**Conclusions:**

This study suggests that the use of zolpidem might increase the risk of subsequent glaucoma. Further confirmatory studies are recommended to clarify this important issue.

## INTRODUCTION

Insomnia is a quite common and distressing problem in the general population. Researchers used to define insomnia as prolonged sleep latency, reduced sleep efficiency, or more than 3 episodes of sleep disturbance per week.^1^ Glaucoma is the main cause of blindness,^2^ and systemic findings in glaucoma patients include alterations of the cardiovascular system, autonomic nervous system, and immune system, as well as endocrinological, psychological, and sleep disturbances.^3^ One study demonstrated a self-reported prolonged sleep-onset latency in normal-tension glaucoma (NTG) patients with vascular dysregulation.^4^ However, the association between glaucoma and insomnia has been well noted but not extensively studied to date.

Zolpidem is a non-benzodiazepine hypnotic drug that enhances GABA(A) receptor function by interaction with the Omega-1 receptor subtype.^5^ It has rapid onset of action, improves total sleep duration, and reduces incidence of night-time awakenings.^5^ Zolpidem is the most commonly prescribed hypnotic agent worldwide and remains the market leader in Taiwan.^6^ Therefore, even a minor hazard could have important clinical implications.^6^

To our knowledge, no published reports have addressed the relationship between zolpidem use and the risk of glaucoma. Here, we gather information and data from Taiwan’s National Health Insurance Research Database (NHIRD) to help us clarify this important issue.

## MATERIALS AND METHODS

### Data source

The Taiwanese government organized the Taiwan health insurance program, which has provided universal and single-payer insurance since 1996 and involves approximately 99% of the 23 million Taiwanese. The National Health Research Institutes (NHRI) established the NHIRD, which contained the annual original claim data for reimbursement. Before NHIRD data was released for research, all personal identification information was encoded to guard patient privacy. This study was approved by the Institutional Review Board of the China Medical University Hospital (CMU-REC-101-012).

The data of the study was collected from the Longitudinal Health Insurance Database (LHID), which is a part of the NHIRD. The LHID contains 1 million insured individuals randomly selected from the NHIRD insured individuals during the period of 1996–2000. According to the NHRI report, there were no differences in age and sex distribution between the NHIRD and LHID. The LHID provided each individual’s information, including sex, birth date, and registry of medical services, and combined these data using a scrambled, anonymous identification number.

Diseases were recorded in the NHIRD using the International Classification of Diseases, Ninth Revision, Clinical Modification (ICD-9-CM). In this study, the disease history and drug usage information were collected from inpatient and outpatient files.

### Study population

This study was a population-based case-control study design. The case group comprised patients newly diagnosed with glaucoma (ICD-9-CM 365) from 2001 to 2010 and set the index date as the date of glaucoma diagnosis. The control group comprised individuals without glaucoma in LHID who were randomly frequency matched at 1:4 ratios by age (in strata of 5 years) and sex. The index date of the control group was a randomly assigned day and month with the same index year as the case group. Both study groups excluded individuals who were prescribed benzodiazepines before the index date.

The risk exposure of interest was zolpidem use. The study also measured the average dose of zolpidem (mg/years), which was defined as the total amount of zolpidem used before the index date divided by the number of years from initial zolpidem use to the index date. Not only demographic factors but also comorbidities were considered as confounding factors. The comorbidities included diabetes (ICD-9-CM 250), coronary artery disease (CAD; ICD-9-CM 410–414), hypertension (ICD-9-CM 401–405), hyperlipidemia (ICD-9-CM 272), anxiety (ICD-9-CM 300.0, 300.2, 300.3, 308.3 and 309.81), and depression (ICD-9-CM 296.2, 296.3, 300.4 and 311) before the index date.

### Statistical analysis

We calculated means and standard deviation (SD) for age and number and proportion for sex, zolpidem use, and comorbidities to describe the distribution of study groups. To test the differences between these two groups, we used the *t*-test for continuous variable and the chi-square test for categorical variables. The odds ratios (ORs) with 95% confidence intervals (CIs) were estimated to examine the potential risk factors’ effect on glaucoma by using the multiple logistic regression method. To understand if the impact of zolpidem use on the risk of glaucoma was moderated by comorbidities, we used the multiple logistic regression method with interaction terms incorporated into the model.

The data management and statistical analysis were performed by SAS 9.3 (SAS Institute, Cary, NC, USA). The statistical significance was defined as (two-sided) *P*-value <0.05.

## RESULTS

There were 8898 glaucoma patients in the case group and 35 592 subjects in the control group. Due to the 1:4 matched-case-control design (by age and sex), as shown in Table 1, subjects in both groups had the same mean age (40.5 years) and sex ratio (57.2% male). The exposure rate of zolpidem use in the glaucoma group was significantly higher than in the control group (2.8% vs. 2.0%, *P* < 0.0001). The occurrence rates of comorbidities, namely hypertension, diabetes, CAD, hyperlipidemia, and anxiety, in the glaucoma group were significantly higher than in the control group (all *P* < 0.0001, except depression).

**Table 1.** Demographic status and comorbidities of the control group and glaucoma group

Variable	Control group *n* = 35 592 (%)	Glaucoma group *n* = 8898 (%)	*P*-value
Age, years (SD)^a^	40.5 (20.5)	40.5 (20.5)	0.8243
<45	20 396 (57.3)	5099 (57.3)	>0.99
45–65	10 096 (28.4)	2524 (28.4)	
≥65	5100 (14.3)	1275 (14.3)	
Sex			>0.99
Female	15 248 (42.8)	3812 (42.8)	
Male	20 344 (57.2)	5086 (57.2)	
Zolpidem exposure			
No	34 892 (98.0)	8646 (97.2)	<0.0001
Yes	700 (2.0)	252 (2.8)	
Zolpidem average exposure, mg/year			
No	34 892 (98.0)	8646 (97.2)	<0.0001
<40	264 (0.7)	81 (0.9)	
40–199	203 (0.6)	73 (0.8)	
≥200	233 (0.7)	98 (1.1)	
Comorbidity			
Hypertension	4491 (12.6)	1622 (18.2)	<0.0001
Diabetes	1754 (4.9)	995 (11.2)	<0.0001
CAD	1337 (3.8)	486 (5.5)	<0.0001
Hyperlipidemia	2672 (7.5)	1176 (13.2)	<0.0001
Depression	110 (0.3)	30 (0.3)	0.6721
Anxiety	224 (0.6)	97 (1.1)	<0.0001

CAD, coronary artery disease; SD, standard deviation.

^a^*t*-test.

After adjusted for hypertension, diabetes, CAD, hyperlipidemia, depression, and anxiety, individuals with zolpidem use had a 1.19-fold increased risk of glaucoma compared to those without zolpidem use (OR 1.19, 95% CI 1.02–1.38; Table 2). Compared to individuals without zolpidem use, the risk of glaucoma was significantly increased in individuals receiving the highest average dose (≥200 mg/year), with an OR of 1.31 (95% CI 1.03–1.68). The results also revealed an increasing trend of glaucoma risk with increasing average dose of zolpidem use (*P*-trend = 0.01).

**Table 2.** Odds ratios^a^ for glaucoma in individual with zolpidem exposure relative to those without zolpidem exposure

Variable	Crude Odds Ratio (95% CI)	Adjusted Odds Ratio (95% CI)
Zolpidem exposure		
No	ref	ref
Yes	1.45 (1.26–1.68)	1.19 (1.02–1.38)
Zolpidem average exposure (mg/year)		
No	ref	ref
<40	1.24 (0.96–1.59)	1.06 (0.83–1.37)
40–199	1.45 (1.11–1.90)	1.19 (0.91–1.57)
≥200	1.70 (1.34–2.15)	1.31 (1.03–1.68)
	*P*-trend < 0.0001	*P*-trend = 0.0120
Hypertension	1.54 (1.45–1.64)	1.16 (1.08–1.25)
Diabetes	2.43 (2.24–2.64)	1.95 (1.77–2.14)
CAD	1.48 (1.33–1.65)	0.98 (0.87–1.10)
Hyperlipidemia	1.88 (1.74–2.02)	1.35 (1.24–1.48)
Depression	1.09 (0.73–1.64)	0.77 (0.51–1.18)
Anxiety	1.74 (1.37–2.21)	1.48 (1.16–1.90)

CAD, coronary artery disease; CI, confidence interval.

^a^Model was adjusted for hypertension, diabetes, CAD, hyperlipidemia, depression, and anxiety.

Table 3 demonstrates the interaction effects between zolpidem use and comorbidities on the risk of glaucoma. These results demonstrated that only diabetes had interaction for risk of glaucoma with zolpidem use (*P* = 0.025).

**Table 3.** Interaction between zolpidem exposure and comorbidities associated with glaucoma risk

Variable		OR (95% CI)	*P*-value^a^
Zolpidem exposure	Hypertension		0.085
No	No	ref	
No	Yes	1.54 (1.45–1.65)	
Yes	No	1.45 (1.20–1.75)	
Yes	Yes	1.72 (1.37–2.16)	
Zolpidem exposure	Diabetes		0.025
No	No	ref	
No	Yes	2.46 (2.26–2.67)	
Yes	No	1.41 (1.20–1.67)	
Yes	Yes	2.29 (1.67–3.15)	
Zolpidem exposure	CAD		0.391
No	No	ref	
No	Yes	1.47 (1.32–1.64)	
Yes	No	1.43 (1.22–1.68)	
Yes	Yes	1.76 (1.23–2.53)	

Zolpidem exposure	Hyperlipidemia		0.101
No	No	ref	
No	Yes	1.88 (1.74–2.03)	
Yes	No	1.40 (1.18–1.67)	
Yes	Yes	2.00 (1.53–2.63)	

Zolpidem exposure	Anxiety		0.134
No	No	ref	
No	Yes	1.79 (1.36–2.35)	
Yes	No	1.44 (1.24–1.68)	
Yes	Yes	1.65 (1.01–2.71)	

Zolpidem exposure	Depression		0.219
No	No	ref	
No	Yes	1.16 (0.73–1.85)	
Yes	No	1.48 (1.27–1.71)	
Yes	Yes	0.94 (0.41–2.15)	

CAD, coronary artery disease; CI, confidence interval; OR, odds ratio.

^a^*P*-value for interaction.

## DISCUSSION

In the present study, we assessed the association between the use of zolpidem and increased risk of glaucoma using a population-based case-control study design. Regarding the effect of zolpidem dosage on the onset of glaucoma, we used zolpidem dosage/per year to evaluate both the total amount of zolpidem used as well as the exposure period. We found that zolpidem dosage was positively correlated to glaucoma risk (*P*-trend < 0.0001). Two potential reasons for this result may be considered: an indirect or direct relationship between zolpidem use and risk of glaucoma.

As far as we know, zolpidem is primarily effective in inducing sleep with minimal effect on sleep duration or sleep maintenance.^7^ The problem of sleep disturbance in glaucoma has been noted previously.^3^^,^^4^ It was proposed that glaucoma patients might have difficulties preparing physiologically for sleep due to an impaired ability to initiate distal vasodilation.^8^ In one study, they found that the NTG patients revealed a prolonged sleep-onset latency both in the evening as well as after nocturnal sleep interruption when rated with a sleep questionnaire.^4^ Flammer et al. reported that glaucoma patients with vasospastic syndrome often suffer from cold hands and cold feet and respond to cold stimuli or even emotional stress with inappropriate constriction or insufficient dilation in the microcirculation.^9^ Systemic vascular dysregulation has been well-noted in glaucoma patients.^10^^,^^11^ A possible explanation for the strong association of zolpidem use and glaucoma might be due to this vascular dysregulation in insomnia patients, which is supported by results of our study.

Another potential direct explanation for the strong association between glaucoma and zolpidem usage is the effect of the drug itself. Zolpidem is mainly metabolized by cytochrome P450 enzymes of the liver, and drug bioavailability can be determined from blood and urine samples.^12^ The possible vascular side effects of zolpidem have been reported in the literature. A large population-based case-control study from Taiwan found that zolpidem exposure was associated with increased risk of ischemic stroke.^6^ Vascular headache associated with hallucination was also noted as an adverse effects of Zolpidem.^13^ In one double-blind and placebo-controlled study assessing zolpidem’s effects within the brain, the dose-related effect of zolpidem in the visual cortex was clarified.^14^ The signal of blood oxygen level-dependent functional magnetic resonance imaging (BOLD fMRI) was significantly reduced at elevated dosages of zolpidem.^14^ To our knowledge, glaucomatous damage is a slowly progressive neuronal degenerative process along the visual pathway.^15^^–^^17^ Decreased cerebral and ocular blood flow as well as impaired vascular autoregulation have been identified in glaucoma patients.^15^ Furthermore, visual cortex involvement in glaucoma patients has also been well studied.^16^^,^^17^ Given these findings, it seems that, for some patients, glaucomatous damage may be not only an ocular manifestation but also indicative of a more widespread vascular abnormality involving the brain.^15^ To identify the direct influence of zolpidem use and risk of glaucoma based on the current results and established theories, further study should be designed to clarify the effect of zolpidem on ocular or cerebral blood flow in the near future.

Another interesting result obtained from the current work is that only diabetes had interaction for risk of glaucoma with zolpidem use (*P*-interaction = 0.025) but not hypertension, hyperlipidemia, or CAD. Several large-scale epidemiologic studies have tried to elucidate the role of metabolic syndrome in glaucoma. In one recent study,^18^ the authors report that those with diabetes (hazard ratio [HR] 1.35, 95% CI 1.21–1.50) or hypertension (HR 1.17, 95% CI 1.13–1.22) alone or in combination (HR 1.48, 95% CI 1.39–1.58) had an increased hazard of developing open-angle glaucoma (OAG) relative to persons without these conditions. In contrast, those with hyperlipidemia alone had a 5% decreased hazard of OAG (HR 0.95, 95% CI 0.91–0.98). We believe different study designs in other ethnic groups might have different findings. Further prospective and longitudinal observational studies are needed to clarify this important issue.

In the face of increasing usage of zolpidem and increasingly reported side effects worldwide, Taiwan’s healthcare community has implemented a strict monitoring system for zolpidem prescription, which creates a virtual private network to remind clinicians of the amount of zolpidem prescribed to each patient over the past 3 months on each visit.^19^ Taiwan residents may also access the Taiwan Consumer Drug Safety platform to read drug safety information for each drug.^20^ All of these policies aim to regulate the usage of hypnotics to prevent drug abuse and related side effects.

We believe that our work has some strengths. The database used is an accurate representative sample population of Taiwanese, and our sample randomization was successful. Furthermore, this dataset captures data on a broad range of subjects of different sociodemographic profiles, unlike some smaller studies that recruit patients from a specific region, which might not represent the whole population. However, some limitations exist in this study. First, the nominal diagnosis of glaucoma and all of the comorbidities that we assessed are all derived from ICD-9-CM diagnosis codes and may be less accurate than diagnoses obtained through a standard procedure. Second, some other potential confounding factors, which could have contributed to zolpidem use and/or glaucoma risk, such as socioeconomic status,^21^ educational background, emotional handicap,^22^^–^^24^ and glaucoma family history,^24^ are not available in our database, which might bias the current work. In one recent study in Canada,^21^ the authors provided evidence that socioeconomic deprivation is associated with greater severity of glaucoma at presentation than those not experiencing socioeconomic deprivation. Glaucoma has been noted to be associated with anxiety, depression, and type-A personality,^22^^,^^23^ and family history of the disease is also a well-known risk factor.^24^ All of these risk factors merit consideration in future research. Third, causality could not be established from this population-based observational longitudinal study because we could not verify the exact temporal relationship between zolpidem use and glaucoma from the database. Fourth, the glaucoma incidence from this database is approximately 0.15% based on the ICD-9 coding 365.xxx, which resulted in a relatively small sample size. To date, there has been no large-scale glaucoma survey in Taiwan. Consequently, the validity of the current database is hard to evaluate without reference data. Furthermore, our current work is the first study to evaluate the relationship between zolpidem use and onset of glaucoma. We do not have any former studies with which to compare our findings.

In conclusion, this population-based case-control study revealed that zolpidem use was associated with a significantly increased risk of glaucoma. In light of the widespread prescription of zolpidem and increasing prevalence of glaucoma, all practicing physicians should bear in mind that glaucoma risk in insomnia patients might increase while taking zolpidem. Further larger confirmatory studies are warranted to clarify this important issue.
